# Structural and functional-annotation of an equine whole genome oligoarray

**DOI:** 10.1186/1471-2105-10-S11-S8

**Published:** 2009-10-08

**Authors:** Lauren A Bright, Shane C Burgess, Bhanu Chowdhary, Cyprianna E Swiderski, Fiona M McCarthy

**Affiliations:** 1 Department of Clinical Sciences, College of Veterinary Medicine, Mississippi State University, PO Box 6100, Mississippi State, MS, 39762, USA; 2 Institute for Digital Biology, Mississippi State University, MS 39762, USA; 3 Department of Basic Sciences, College of Veterinary Medicine, Mississippi State University, PO Box 6100, Mississippi State, MS, 39762, USA; 4 Mississippi Agricultural and Forestry Experiment Station, Mississippi State University, MS 39762, USA; 5 Department of Veterinary Integrative Biosciences, College of Veterinary Medicine & Biomedical Sciences, Texas A&M University, USA

## Abstract

**Background:**

The horse genome is sequenced, allowing equine researchers to use high-throughput functional genomics platforms such as microarrays; next-generation sequencing for gene expression and proteomics. However, for researchers to derive value from these functional genomics datasets, they must be able to model this data in biologically relevant ways; to do so requires that the equine genome be more fully annotated. There are two interrelated types of genomic annotation: structural and functional. Structural annotation is delineating and demarcating the genomic elements (such as genes, promoters, and regulatory elements). Functional annotation is assigning function to structural elements. The Gene Ontology (GO) is the *de facto *standard for functional annotation, and is routinely used as a basis for modelling and hypothesis testing, large functional genomics datasets.

**Results:**

An Equine Whole Genome Oligonucleotide (EWGO) array with 21,351 elements was developed at Texas A&M University. This 70-mer oligoarray was designed using the approximately 7× assembled and annotated sequence of the equine genome to be one of the most comprehensive arrays available for expressed equine sequences. To assist researchers in determining the biological meaning of data derived from this array, we have structurally annotated it by mapping the elements to multiple database accessions, including UniProtKB, Entrez Gene, NRPD (Non-Redundant Protein Database) and UniGene. We next provided GO functional annotations for the gene transcripts represented on this array. Overall, we GO annotated 14,531 gene products (68.1% of the gene products represented on the EWGO array) with 57,912 annotations. GAQ (GO Annotation Quality) scores were calculated for this array both before and after we added GO annotation. The additional annotations improved the *meanGAQ *score 16-fold. This data is publicly available at *AgBase *http://www.agbase.msstate.edu/.

**Conclusion:**

Providing additional information about the public databases which link to the gene products represented on the array allows users more flexibility when using gene expression modelling and hypothesis-testing computational tools. Moreover, since different databases provide different types of information, users have access to multiple data sources. In addition, our GO annotation underpins functional modelling for most gene expression analysis tools and enables equine researchers to model large lists of differentially expressed transcripts in biologically relevant ways.

## Background

Although the availability of a completed horse genome sequence enables researchers to use genomic technologies in their research [1], deriving value from high throughout genomics datasets requires genomic annotation. Genomic annotation includes the demarcation of functional elements within the genomic sequence ("structural annotation") and associating functional data with these same elements ("functional annotation"). Structural annotation is initially provided during the final stages of genome sequence assembly using computational pipelines to predict open reading frames and other functional elements. For example, the National Center for Biotechnology Information (NCBI) Gnomon annotation pipeline http://www.ncbi.nlm.nih.gov/genome/guide/gnomon.shtml combines ab initio predictions with sequence homology based upon RefSeq transcript alignments of the known genes. This structural annotation pipeline currently identifies 21,842 horse genes, and of these, 82.4% are "predicted" based upon sequence similarity with known genes from other species (as of 10/04/08). This means that these 17,997 horse genes are only listed because they are similar in sequence to genes that are already known to exist in other species.

In contrast to structural annotation, functional annotation is not generally done automatically as part of the genome sequencing process. Typically, functional annotation is done as a separate, focused effort and the de facto method for functional annotation in eukaryote genomes is the Gene Ontology [2]. The GO is a structured network consisting of defined terms and the relationships between them that describe three attributes of gene products: Molecular Function, Biological Process and Cellular Component [3]. Annotation to the GO involves providing information about the gene product being annotated, its attributed function and the evidence for associating the function with this gene product [4]. There are two broad types of GO evidence codes: direct experimental codes (the evidence codes used for biocuration of published literature) and indirect evidence codes. Indirect evidence codes include function prediction based on sequence such as "inferred from sequence orthology" (ISO), where functional conservation is inferred for predicted orthologs, and "inferred from electronic annotation" (IEA), which includes function predicted based on functional motifs and domains [5]. The European Bioinformatics Institute GOA Project (EBI GOA) provides IEA based GO annotations for all proteins in the UniProtKB database [5].

Analyzing microarray data using GO has provided new insights into agriculturally important areas of research, including reproduction [6], lactation [7], adipogenesis [8] and animal health [9,10]. Moreover, GO annotation has become the accepted standard for functional annotation and its use is growing exponentially in species that have a history of dedicated GO annotation effort [2,11]. GO annotations provided by GO Consortium members are used by public databases (eg. Entrez Gene, UniProt), genome browsers (eg. Ensembl), commercial vendors (eg. Affymetrix, Ingenuity Pathways Analysis) and freely available analysis tools (eg. Onto-Tools [12], Cytoscape [13]). However, while there are many tools available for analyzing microarray data http://www.geneontology.org/GO.tools.shtml#micro, researchers wishing to do functional analysis of their equine array results are hampered by the lack of GO for equine gene products represented on microarrays. For example although there are, 21,842 horse genes, only 1,582 equine proteins are represented in the UniProtKB database, so only 7.2% of horse gene products have any GO annotation. This is further complicated since different tools use different database accessions, and it is currently difficult to determine the equivalent database accessions for horse sequences found in different public databases.

If equine researchers are to translate functional genomics results into practical solutions for equine health and production, they need to be able to translate data provided by high throughput functional genomics platforms (such as microarrays) into relevant biological knowledge. The Texas A&M Equine Whole Genome-oligoarray is a 21,000 element 70-mer expression array designed from the assembled equine genome sequence in order to represent the majority of expressed equine sequences.

Briefly, the vast majority (97.5%) of the genes were documented by one or more transcript sequences (RNA, UniGene or EST) while the remaining (2.5%) were documented solely by a protein hit. The oligo design process searched for 70-mer long hybridization probes representing all genes with due consideration to probes reporting multi-copy genes and other more complex cases. The probe selection process resulted in 21,351 probes (20,461 addressing single-gene and 890 addressing multi-gene targets) representing 22,296 genes. Appropriate positive, distance, specificity and negative controls (total 321) were added. The probes were commercially synthesized (Invitrogen, USA) and spotted onto UltraGAPS aminosilane coated slides with barcodes (Corning, MA) using a Chip Writer Pro microarrayer (BioRad, CA) equipped with 24 Telechem SMP3 pins (TeleChem International, CA) [14,15].

The Equine Whole Genome array is presently being validated and will be available to the equine research community worldwide. To assist equine researchers with the functional modelling of data produced using this array, we provide information about public database accessions and functional annotations for elements represented on this array. The method of functional annotation that we use to provide GO annotation for this array is a combination of manual biocuration with computational analysis. We are continuing to add additional GO annotations based upon published literature and all GO annotations will be made publicly available at the AgBase http://www.agbase.msstate.edu/.

## Results and discussion

Array annotation is useful because it facilitates integrating and interpreting large data sets that are produced when oligoarrays are used to evaluate complex biological processes. By annotating the equine whole genome array, researchers can step from lists of differentially regulated gene products to model-based clustering of gene expression data that advances the understanding of a biologic process. Further, accurate modelling requires up-to-date functional annotation, regardless of species, and is relevant to physiology, health, and disease.

The importance of integrating biological knowledge gleaned from gene expression profiles has been eloquently demonstrated by Chen and Wang. Using breast cancer microarrays, they demonstrated that prediction models constructed based on information from gene sets (pathways) outperformed the prediction models based on expression values of single genes, with improved prediction accuracy and interpretability [16]. This approach has also been applied to investigate the molecular basis of bone remodelling in osteoarthritis. The researchers conducted a microarray gene expression profile of the bone. Through this profile, researchers identified altered expression of two signalling pathways and target genes in osteoarthritic bone. Using an annotated array, these researchers were able to include genes with known or predicted roles in osteoblast, osteocyte, and osteoclast differentiation and function [17].

The Texas A&M Equine Whole Genome-oligoarray is a 21,351 element expression array that is presently being validated and will be available to the equine research community worldwide. To ensure that users are able to derive value from their array results we have provided information about the public database accessions represented on this array and provided GO annotations for these gene products.

### Database accession mapping

So that users could access the information from multiple public databases, we provided multiple database accessions corresponding to each element on the array. To do this we used ArrayIDer [18], a tool that retrieves structural annotations for ESTs and provides 13 different identifiers for access to several publicly available databases (including UniProtKB, Ensembl, RefSeq, IPI and UniGene). An example of the ArrayIDer output is shown (Additional file 1) and the complete results will be made publicly available both with the array and on the *AgBase *website http://www.agbase.msstate.edu/. Until this data is available online, users can contact AgBase for this mapping table or to run ID mapping for datasets.

**Figure 1:**
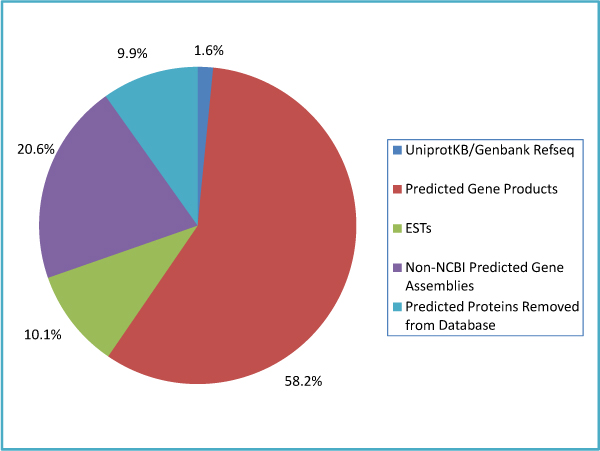
**Gene products represented on the equine whole genome array**. Array gene products were linked to public databases to facilitate functional modelling. 1.6% of the elements represent experimentally validated products found in UniProtKB or the RefSeq databases while 58.2% are predicted based upon computational structural annotation of the horse genome. 20.6% are predicted genes not available from NCBI and 10.1% are ESTs that are not linked to known or predicted horse genes. A further 9.9% have been removed from the NCBI databases due to structural reannotation.

The presence or absence of these gene products in different databases (Figure 1) also provides biological clues about these gene products. For example, we found 337 elements that map to UniProt or Genbank RefSeq accession numbers. These are the equine gene products that were experimentally studied prior to gene sequencing and are likely to have published functional information available. A further 12,343 elements map to "XP" accessions from NCBI; these are proteins that are predicted based upon the NCBI structural annotation pipeline. These predicted gene products will not yet have experimental functional information available but they will have sequence homology to experimentally validated genes other species. The relatively large proportion of predicted gene products is typical of newly sequenced genomes such as horse. For example 84% of equine genes in NCBI are predicted, compared to 57.3% of the gene products represented on this array. Moreover there are 4,399 additional gene assemblies represented on this array that are not available from NCBI.

We found 2,164 Expressed Sequence Tags (ESTs) that did not map to any of the current equine genes. ESTs represent the transcriptionally expressed elements within a genome and since these do not align with any predicted genes, these may represent mRNAs that are unique to horse. Another feature of newly sequenced genomes is that there are rapid revisions and changes to the publicly available gene products as structural annotation proceeds. We found 2,108 elements mapping to NCBI database accessions that have been removed due to updates in structural annotation of the equine genome.

### GO annotation results

**Figure 2:**
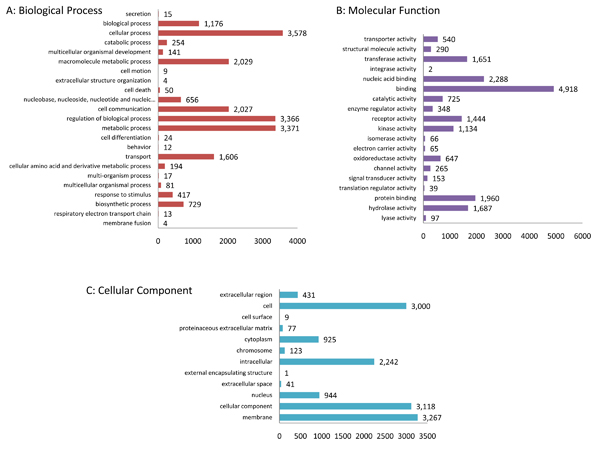
**Functional grouping of equine array gene products using GOSlimViewer**. The GO annotation is divided into three broad functional groups using the GOA and whole proteome GOSlim and the GOSlimViewer tool: A. Biological Process, B. Molecular Function, and C. Cellular Component. Further subcategories within functional groups A-C are listed on the y-axis and the frequency of this function within the array is represented on the x-axis. The functional group, "biological process" had the most GO IDs represented, followed by "molecular function," and finally "cellular component." In A, the largest three subcategories were: cellular process, regulation of biological process, and metabolic process. In B, binding was the most annotated function. For C, the top three cell component subcategories were e cell, cell membrane, and cellular component. Particularly significant is the wide display of GO IDs shown, suggesting the equine whole genome array is fairly comprehensive.

Since the EBI GOA Project provides IEA annotation for UniProtKB proteins, we found that 208 equine UniProtKB entries already had existing GO. This represents 61.4% of the UniProtKB but only 1% of the elements on the array. To improve the amount of GO annotation for horse gene products represented on this array we did our own GO annotation for equine gene products. In total, we added 57,912 GO annotations for 14,531 gene products, representing 68.1% of the elements on the Equine Whole Genome-oligoarray. Using a similar approach, the Affymetrix chicken genome array was reannotated, increasing the number of probes associated with GO annotation by 45% and the quality of annotation by 14%. The large proportion of equine gene products associated with GO is partially due to the improved ability to recognize equine: mammal orthologs (compared to chicken) and that 31.9% of these gene products were listed as "No Data" (ND), indicating that there is presently no functional information for these elements. This GO annotation is summarized into broad functional groups using the GOA and whole proteome GOSlim and the GOSlimViewer tool [19] (Figure 2). The GO annotation is divided into three groups: cellular component, molecular function, and biological process. The GO is evenly represented as 38% of the annotations are biological processes, 35% are molecular functions, and 27% are cellular components. Furthermore, there is information about membranes, cells, binding, regulation of biological processes, cell communication, cellular processes, and metabolic processes, along with much more. Thus annotation allows investigators to rapidly translate and integrate the full complement of array data into a bar code of structurally and functionally meaningful changes at the protein level, changes which reflect the differential regulation of the experimental intervention.

Since UniProtKB and RefSeq accessions are likely to have literature that delineates protein function, we provided GO annotations by manual curation of existing literature. Since this process is necessarily time consuming this effort is continuing. To provide initial GO annotation we used known orthologs to human, mouse and rat genes that have existing GO. Orthologs were manually verified and GO annotation based on direct, experimental evidence transferred to the equine proteins. Fifty gene products were manually annotated using ISO annotation, producing 529 annotations. (See Additional file 2 for a list of the gene products and the 529 correlating annotations.) A further 43 cannot be annotated until confirmation of the existence or absence of any literature available.

While there is no experimental literature for any of the equine predicted proteins, many of these are likely to have orthologs amongst mammalian species. By transferring GO annotations from orthologous genes products that have experimental based GO annotation 48,887 annotations were added for 12,227 predicted proteins, representing 98.3% of all predicted proteins. The other elements on the array had no experimental literature or ortholog information available. Instead we provided GO annotations based upon functional motifs using an automated pipeline to assess functional motifs [20]. We added 6,466 annotations for 4,154 gene products, representing 23.6% of all gene products. Notably, 76.4% of the gene products were annotated as "no data." These are summarized in Table 1.

**Table 1:** GO Annotation of the equine whole genome oligoarray

Database Category	No. Gene Products	Number of GO annotations added
**UniprotKB/Genbank Refseq**	337	2,559
**Predicted Gene Products**	12,434	48,887
**ESTs**	2,164	4,546
**Non-NCBI Predicted Gene Assemblies**	4,399	1,920
**Predicted Proteins Removed from Database**	2,108	--
***TOTAL***	21,351	57,912

A total of 57,912 annotations were derived from the Equine Whole Genome Oligoarray. 2,559 annotations were derived from the 337 UniprotKB and RefSeq accessions. 48,887 annotations were derived from 12,434 predicted gene products. 4,546 annotations were derived from 2,164 ESTs. Finally, 2,108 predicted proteins removed from the database were not annotated. Thus 21,351 gene products yielded 57,912 new annotations.

Currently there are no commercial equine arrays, so there is no GO associated with any of the other equine arrays. However we do have information about the GO provided for commercial arrays in other livestock species. Notably, although Affymetrix provides GO annotation with their array annotation files, re-annotation of GeneChip Chicken Genome Array resulted in a 37% increase in the number of array elements with GO annotations and a 14% increase in the GO annotation quality [21]. For the more closely related pig, only 11% of gene products on the Affymetrix array have GO annotation.

The GO annotations that we have provided will be made publicly available via the *AgBase *database. Since GO annotations change as new data becomes available and new GO terms are added, this information will be updated periodically. Providing GO annotations for 68% of the elements on the equine array is a significant achievement and work is continuing to provide more detailed GO annotations and make these publically available. Array users are encouraged to contact *AgBase *agbase@cse.msstate.edu with specific questions about this data or to request further GO annotation.

### GAQ score results

**Figure 3:**
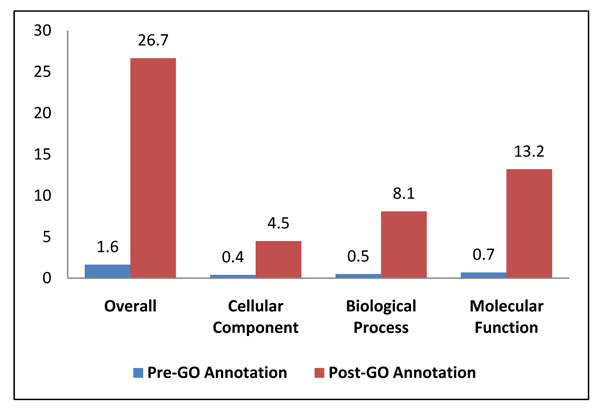
**GO Annotation Quality (*GAQ*) score**. GAQ Scores were calculated for the existing GO annotation on the array and the GO annotation available after we added the additional annotations described in this paper. *GAQ *Scores are calculated exactly as described previously [22]. Briefly, *GAQ *score quantitatively measures GO quality, which includes breadth of GO annotation, the level of detail of annotation (depth), and the type of evidence used to make the annotation. Additional GO improved the *meanGAQ *score 16-fold, from 1.6 for the pre-existing GO to 26.7 for the completed or additional GO. *meanGAQ *score for each ontology is shown as well. Cellular component increased 11-fold, from 0.4 to 4.5, biological process increased 16-fold, from 0.5 to 8.1, and molecular function increased 18-fold, or from 0.7 to 13.2.

To determine the overall quality of the GO annotations added to the array, we evaluated the GO Annotation Quality Score[22] for gene products associated with this array both before and after we added our GO annotations. Briefly, *GAQ *score quantitatively measures GO quality, which includes breadth of GO annotation, the level of detail of annotation (depth), and the type of evidence used to make the annotation. *GAQ *Scores are calculated exactly as described previously [22] and the meanGAQ Score, the average GAQ Score for the dataset reported (Figure 3). Our GO annotations improved the *meanGAQ *score 16-fold for the array, from 1.6 for the pre-existing GO to 26.7 for the completed or additional GO. The *meanGAQ *score was also reported and as expected there was an increase for each of the three ontologies. Cellular component increased 11-fold, from 0.4 to 4.5, biological process increased 16-fold, from 0.5 to 8.1, and molecular function increased 18-fold, or from 0.7 to 13.2.

## Conclusion

This work is an initial computational based survey to provide GO annotation for a broad range of equine gene products. However detailed, species specific function can only be derived from manual curation of experimental literature and necessarily requires a focused biocuration effort which is currently lacking for horse. Nevertheless, this GO annotation provides the overview required to facilitate functional modelling of equine datasets based upon this array. Moreover, the GO annotations are made publicly available and will assist all equine researchers wishing to use the GO to model their data.

## Methods

### Accession mapping

Accession mapping was done using the standalone version of ArrayIDer from AgBase[18]. ArrayIDer accepts data from any microarray containing expressed sequence tag (EST) identifiers compatible with the NCBI UniGene database. ArrayIDer generates a list of gene and protein accessions from the latest databases (NCBI UniGene and the International Protein Index) and retrieves identifiers that match the EST input list. ArrayIDer will be activated for the horse dataset but until this is available online, users may contact AgBase to retrieve the mapping table or to run accession mapping for their own datasets. ArrayIDer is available from *AgBase *http://www.agbase.msstate.edu/arrayider.html.

### GO annotation

**Figure 4:**
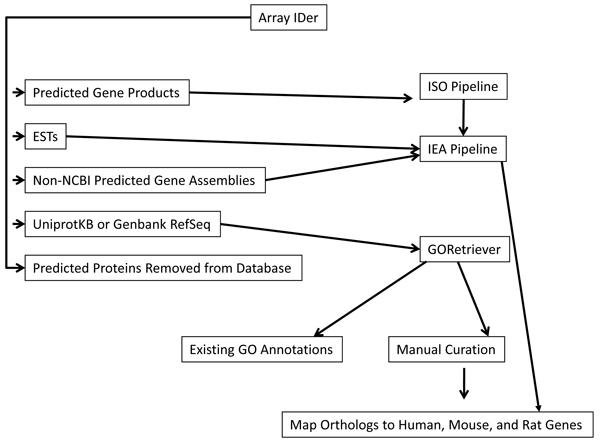
**Flow chart demonstrating the functional annotation process**. Functional annotation begins by accession mapping through ArrayIDer. ArrayIDer divides the input file into broad categories: predicted gene products, ESTs, non-NCBI predicted gene assemblies, and UniprotKB or Genbank RefSeq, as well as predicted proteins that were removed from the database. Predicted gene products go down the ISO pipeline, and the rest go through IEA pipelines, with the exception of UniprotKB or RefSeq, which are sent to GORetriever. GORetriever pulls out the genes which already have existing GO annotations, and the rest are manually curated by mapping orthologs to human, mouse, and rat genes.

Our strategy for providing GO annotations for gene products represented on this array is summarized in Figure 4. We initially used GORetriever [19] to determine which UniProtKB or RefSeq accessions already had existing GO annotations. The remaining UniprotKB and RefSeq accessions were manually GO annotated based upon functional literature and mapped to orthologous mammalian gene products with experimentally based GO. Orthologs were determined using Ensembl version 53 and only 1:1 orthologs from human, rat, or mouse were returned. This type of GO annotation was assigned "inferred from sequence orthology" (ISO) GO evidence code, based upon standard GO Consortium procedures [4].

The NCBI predicted proteins do not have direct experimental evidence, and are unlikely to have any orthologs. These were first annotated by ISO annotation, or if there was no 1:1 ortholog available, we used known functional motifs to provide GO annotation. This is an automated process and is referred to as "inferred from electronic annotation" (IEA). Other gene products represented on the array were also GO annotated using the IEA method.

The results of these GO annotations were summarized using GOSlimViewer [19] with the GOA and whole proteome GOSlim Set.

## List of abbreviations used

EBI-GOA: European Bioinformatics Institute GOA Project; ESTs: Expressed Sequence Tags; EWGO: Equine Whole Genome Oligoarray; GAQ: GO Annotation Quality; GO: Gene Ontology; IEA: Inferred from Electronic Annotation; ISO: Inferred from Sequence Orthology; NCBI: National Center for Biotechnology Information; ND: No Data; NRPD: Non Redundant Protein Database.

## Competing interests

The authors declare that they have no competing interests.

## Authors' contributions

LB provided manual GO annotations for this manuscript and contributed to aspects of the manuscript preparation. CS and SCB initiated the project and assisted with manuscript preparation. BC developed the equine array and provided the details and sequences associated with this array. FM co-ordinated the project, collated the annotation data and prepared the manuscript. All authors read and approved the final manuscript.

## Supplementary Material

Additional file 1 **ArrayIDer Output**. To facilitate linking array data to information in multiple public databases, ArrayIDer retrieves structural annotations for array elements and provides corresponding identifiers used in public databases (including UniProtKB, Ensembl, RefSeq, IPI and UniGene). The identifiers are: probe name, horse gene ID, the public accession number, the Unigene ID, any gene symbols it has, the Entrez Gene ID, its RefSeq accession number, and its UniprotKB ID. This is only an example, and the rest of the equine array data from ArrayIDer will be made publicly available via *AgBase*. Click here for file

Additional file 2 **List of the fifty manual curated genes and respective GO annotations**. This excel file is a list of the genes that were manually curated. File includes probe ID, database, accession number, and name of gene. The file also includes the 529 annotations that correlate to the fifty genes. Click here for file
